# Low Temperature Powder Bed Fusion of Polymers by Means of Fractal Quasi-Simultaneous Exposure Strategies

**DOI:** 10.3390/polym14071428

**Published:** 2022-03-31

**Authors:** Samuel Schlicht, Sandra Greiner, Dietmar Drummer

**Affiliations:** 1 Institute of Polymer Technology, Friedrich-Alexander-Universität Erlangen-Nürnberg, Am Weichselgarten 10, 91058 Erlangen, Germany; sandra.greiner@fau.de (S.G.); dietmar.drummer@fau.de (D.D.); 2 Collaborative Research Center 814, Friedrich-Alexander-Universität Erlangen-Nürnberg, Am Weichselgarten 10, 91058 Erlangen, Germany

**Keywords:** powder bed fusion, laser sintering, isothermal, low temperature laser sintering, selective laser melting

## Abstract

Powder Bed Fusion of Polymers (PBF-LB/P) is a layer-wise additive manufacturing process that predominantly relies on the quasi-isothermal processing of semi-crystalline polymers, inherently limiting the spectrum of polymers suitable for quasi-isothermal PBF. Within the present paper, a novel approach for extending the isothermal processing window towards significantly lower temperatures by applying the quasi-simultaneous laser-based exposure of fractal scan paths is proposed. The proposed approach is based on the temporal and spatial discretization of the melting and subsequent crystallization of semi-crystalline thermoplastics, hence allowing for the mesoscale compensation of crystallization shrinkage of distinct segments. Using thermographic monitoring, a homogenous temperature increase of discrete exposed sub-segments, limited thermal interference of distinct segments, and the resulting avoidance of curling and warping can be observed. Manufactured parts exhibit a dense and lamellar part morphology with a nano-scale semi-crystalline structure. The presented approach represents a novel methodology that allows for significantly reducing energy consumption, process preparation times and temperature-induced material aging in PBF-LB/P while representing the foundation for the processing of novel, thermo-sensitive material systems in PBF-LB/P.

## 1. Introduction

Laser-based powder bed fusion of polymers (PBF-LB/P) is a powder-based additive manufacturing process that allows for manufacturing individualized components with a high geometric freedom. To date, PBF-LB/P is predominantly associated with the quasi-isothermal processing of semi-crystalline polymers. Given the continuous heating of the build chamber in quasi-isothermal PBF-LB/P, temperature-induced aging of polymers [1], increased process times due to heating and cooling phases, and the influence of processing times on resulting mechanical properties [2,3] inherently restrict the economic and ecological viability for the cost-efficient manufacturing of polymer components. Considering non-uniform temperature fields occurring in PBF-LB/P, the isothermal assumption merely represents an idealization. With regard to isothermal crystallization kinetics of Polyamide 12, findings by Neugebauer et al. [4] indicate the occurrence of isothermal crystallization during isothermal PBF of polymers. Using a process-integrated approach, Drummer et al. [5] determined the time-dependent occurrence of isothermal crystallization in PBF-LB/P, proposing the possibility of novel process strategies for limiting the isothermal processing zone to the powder bed surface. Considering a time- and temperature-dependency of the isothermal crystallization process, findings by Soldner et al. [6] indicate a non-uniform, geometry-dependent isothermal crystallization [7]. Findings derived by Shen et al. [8] based on a numerical approach indicate a correlation of the underlying cooling rate and resulting residual stress, thus affecting part distortion. Consequently, controlled isothermal crystallization is omnipresent in PBF-LB/P, being influenced by time-, temperature-, and geometry-dependent effects. However, even considering the occurrence of isothermal crystallization in PBF-LB/P, the non-isothermal processing below the crystallization onset temperature of semi-crystalline polymers remains restricted due to the occurrence of stress-induced distortion, specifically curling and warping [9]. Induced by the inhomogeneous crystallization of the polymer melt, curling considerably reduces the process stability, leading to process interruptions. Crystallization-induced shrinkage is inherently bound to the processing of semi-crystalline polymers, thus constituting the requirement of novel strategies for controlling the crystallization kinetics for promoting a uniform, controlled crystallization of each layer. Therefore, the non-uniform crystallization of the applied materials constitutes an inherent challenge for novel processing strategies, focusing on the exposure-induced process optimization.

## 2. State of the Art

### 2.1. Kinetics of Isothermal and Non-Isothermal Crystallization of Polymers

Quasi-isothermal processing composes the state of the art in laser-based powder bed fusion of polymers. The isothermal assumption in PBF-LB/P implies the predominant occurrence of isothermal crystallization during the build process. A basic modelling of isothermal crystallization processes can be derived using the Avrami equation. Considering non-constant cooling rates, occurring in laser sintering of polymers in quasi-isothermal as well in non-isothermal processing, the Nakamura model, proposed by Nakamura et al. [10,11] allows for considering non-isothermal crystallization. The macroscopic degree of crystallization, *α*, can be expressed in dependence of the Nakamura kinetics crystallization function *K*(*T*), being closely related to the Avrami function *k*(*T*).

(1) $$\alpha =1-\exp\left[-{\left(\int_{0}^{t}K\left(t\right)dt\right)}^{n}\right]$$

The underlying relation of the Nakamura crystallization rate *K*(*t*) and the Avrami crystallization rate *k*(*t*) can be expressed using a temperature-dependent, dimensionless parameter *n*.

(2) $$K\left(t\right)=k{\left(t\right)}^{\frac{1}{n}}$$

Ziabicki [12] described an empirical exponential relation of the crystallization half time *t*_1/2_, the growth constant *K*_0_ and the nucleation rate constant *K_g_* by applying the Lauritzen–Hoffman theory, with the activation energy for polymer diffusion U* = 6270 J mol^−1^, the universal gas constant *R*, the temperature value *T_∞_* = *T_g_* − 30 K, indicating a ceased viscous flow, and the equilibrium melting temperature $T_{m}^{0}$, displayed in Equation (3).

(3) $$\frac{1}{t_{1/2}}{=K}_{0}\exp\left[-\frac{U^{*}}{R\left(T-T_{\infty }\right)}\right]\;\exp\left[\frac{K_{g}{(T+T}_{m}^{0})}{{2T}^{2}{(T}_{m\;}^{0}-T)}\right]$$

Zhao et al. [13] applied the empirical relation on Polyamide 12, used in laser-based powder bed fusion, by fitting the parameters *K*_0_ and *K_g_* based on experimental data obtained from differential scanning calorimetry. The resulting crystallization half times, presented by Zhao et al., exhibit a satisfactory accordance of experimentally obtained and modelled values for sufficiently high cooling rates. Consequently, a reduced processing temperature *T* is correlated with considerably reduced crystallization half times. Considering isothermal crystallization of quenched Polyamide 12 at varying ambient temperatures, Paolucci et al. [14] modelled the temperature-dependent formation of varying crystalline phase compositions of Polyamide 12. Unpressurized crystallization at temperatures exceeding 100 °C predominantly leads to the formation of the α-phase [14], implying the formation of an identical crystalline phase within a wide thermal processing window. However, with regard to described crystallization kinetics, a dependency of the applied cooling rate and morphological properties needs to be considered a major influence in low temperature PBF-LB/P.

### 2.2. Low Temperature Laser-Based Processing of Polymers

In contrast to powder bed fusion of metal alloys, PBF of polymers is characterized by the avoidance of support structures. With regard to the application of support structures, different approaches for transferring metal-based concepts on the non-isothermal processing of polymers [15] have been described. Resulting material morphologies exhibit significantly reduced spherulite sizes, correlated with an increased elongation at break while exhibiting an insufficient porosity level [16]. In contrast to the application of support structures, the avoidance of curling and warping by means of laser-based preheating has been proposed by Laumer et al. [17], applying simultaneous laser beam irradiation for the isothermal manufacturing of multi-material components. Investigations conducted by Chatham et al. [18] for the manufacturing of polyphenylene sulfide at reduced powder bed temperatures exhibit significantly increased levels of porosity, thus limiting the applicability of produced parts. Consequently, to date, the non-isothermal PBF of polymers is inherently limited with regard to the requirement of support structures and the emergence of insufficient part properties.

### 2.3. Influence of Exposure Strategies on Superficial Temperature Fields and Part Properties

The application of a variety of exposure strategies has been described for both metal-based and polymer-based PBF. The interaction of an exposed geometry and the applied exposure strategy on resulting temperature fields is described extensively in recent literature. Exhibiting a reduced thermal penetration depth, increased exposure speeds are correlated with increased superficial maximum temperature values [19,20]. In addition, resulting superficial temperature fields show a dependence on both the applied exposure speed and the underlying scan vector length [19], resulting from a thermal superposition of subsequently exposed scan vectors [20]. Jain et al. [2] describe a correlation of geometry-induced, varying return times of the laser beam and varying mechanical properties of samples manufactured using Polyamide 12, implying an influence of superficial temperature fields on resulting mechanical part properties. Exceeding the monitoring of exposure-induced temperature fields, Greiner et al. (2021) [21] observed an interdependence of applied exposure parameters and the underlying geometry on post-exposure temperature fields, leading to a varying morphological structure of fabricated parts.

Segmented exposure strategies, widely applied in laser-based PBF of metal alloys (PBF-LB/M), exhibit reduced residual stresses, described for the application of steel [22,23,24] and nickel alloys [25]. Zou et al. (2020) [22] describe a significant influence of the exposure sequence and orientation of distinct segments on resulting residual stress, emphasizing structural advantages of non-linear sequencing compared to linear sequencing. Considering varying exposure patterns of distinct segments, further complex interdependencies of the applied sequence of exposed scan vectors, the scan vector length and the sequence of exposed sub-segments are described. With regard to the inherent geometry-dependence of linear exposure patterns, non-linear exposure patterns are gaining increased attention both in metal- and polymer-based PBF. The application of non-linear exposure patterns was initially described by Yang et al. [26], applying the space-filling, fractal Hilbert curve for the sintering of polymer-bound ceramic particles. Further research on fractal exposure patterns was conducted by Ma et al. [27] and Catchpole-Smith et al. [28], describing reduced stress-induced distortion and the reduced occurrence of heat-induced cracks in PBF of nickel alloys. Greiner et al. [29] described the application of the fractal, space-filling Peano curve for the PBF of Polyamide 12, leading to geometry-invariant temperature fields promoted by the scale-invariant structure of the applied exposure pattern. Therefore, the application of linear exposure patterns is considerably influenced by the exposed cross-section, leading to a reduced reproducibility of part properties. In contrast, applying segmented, fractal exposure strategies promote the formation of uniform, geometry-invariant temperature fields that could be exploited for low temperature PBF-LB/P. Consequently, the formation of crystallization-induced residual stress and resulting part deformations exhibits a dependence of applied exposure strategies, hence implying the requirement of novel exposure strategies to overcome existing limitations of quasi-isothermal PBF of polymers.

## 3. Methodological Approach for Low Temperature PBF

**Figure 1:**
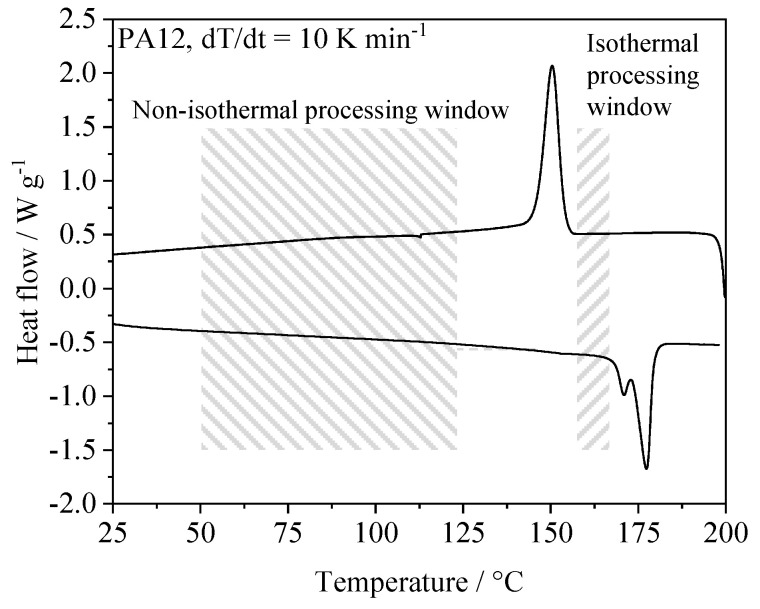
Schematic illustration of process-dependent thermal processing windows.

The approach presented in this paper focusses on significantly lowering the build chamber temperature while limiting warping and curling of manufactured parts by means of fractal, quasi-simultaneous laser exposure. In contrast to quasi-isothermal PBF, low temperature PBF, as proposed in this paper, relies on the immediate crystallization of distinct exposed segments, considering a material-specific processing windows below the crystallization peak, displayed in Figure 1.

**Figure 2:**
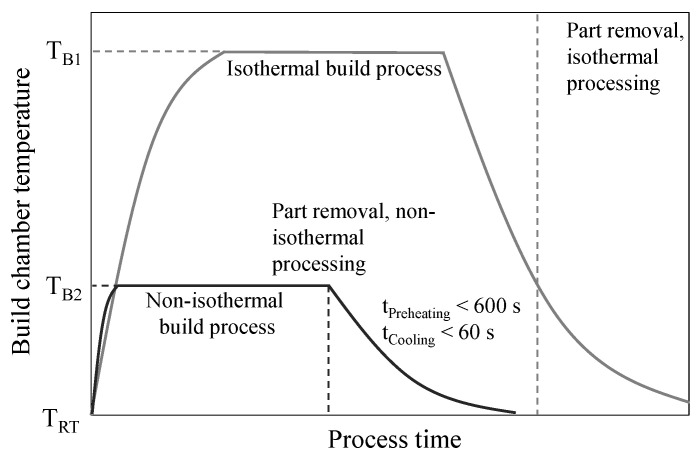
Schematic time-dependent temperature variation for applying quasi-isothermal and non-isothermal processing.

Resulting implications for process temperature control include significantly reduced pre-heating times and the immediate removal of manufactured parts subsequent to the build process. Reduced pre-heating times are obtained considering reduced requirements of the thermal homogeneity in contrast to quasi-isothermal processing, thus limiting the required homogenous thermal field to the thickness of the manufactured layer. Resulting process times of non-isothermal and quasi-isothermal processing, respectively, are displayed in Figure 2.

**Figure 3:**
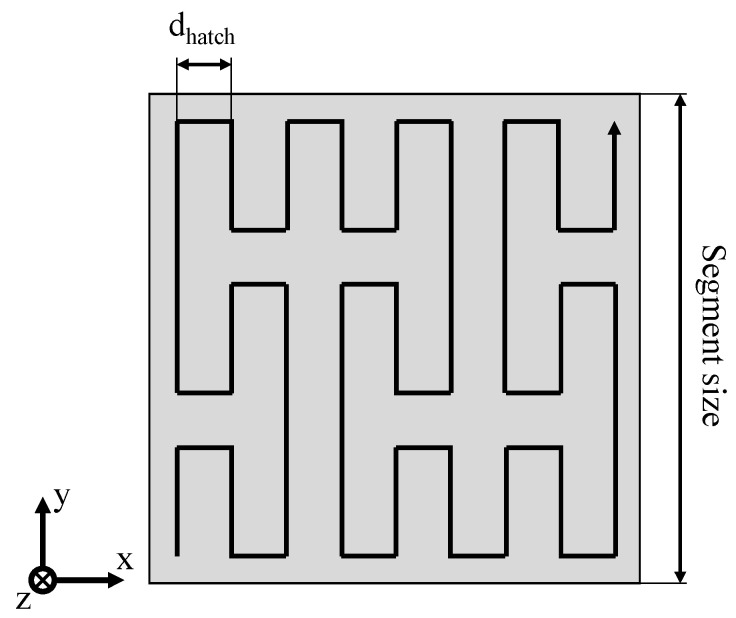
Schematic depiction of the applied fractal exposure pattern.

To allow for the non-isothermal processing of semi-crystalline polymers, restricting the distortion of exposed segments is essential. Based on previous research on the field of segmented exposure strategies [23,25], segmented exposure strategies are combined with the application of fractal scan paths [26,27,28,29] and quasi-simultaneous exposure of distinct segments. Fractal scan path generation applied within the present paper is based on space-filling, self-avoiding and self-similar curves, commonly referred to as “FASS curves” [30], specifically on the Peano curve [31]. The implementation of fractal, quasi-simultaneous exposure strategies is based on functional recursive programming, conducted in Python 3.8. Resulting exposure strategies are transferred using the Common Layer Interface (CLI) format to allow for the integration of complex exposure strategies into commercially available machinery. Exposure paths, applied for the non-isothermal processing of polymers, include discrete fractal sub-segments that are exposed using fractal sequencing. The resulting exposure strategy of an exemplary square cross-section is displayed in Figure 3.

**Figure 4:**
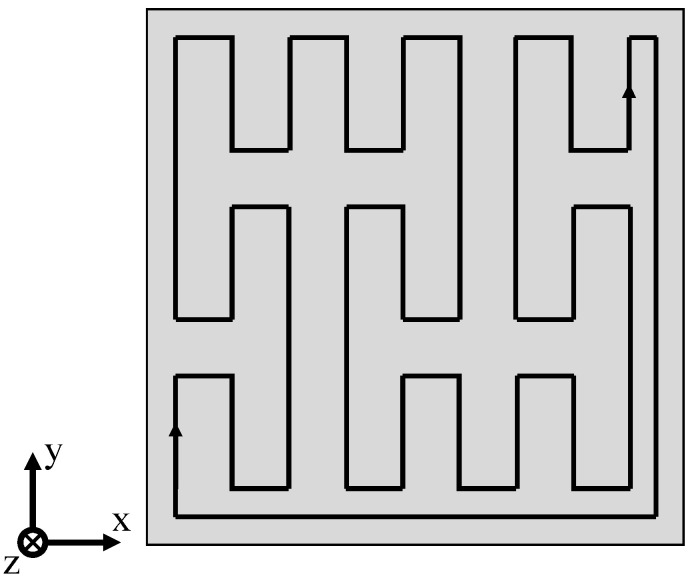
Fractal, quasi-simultaneous exposure pattern of a distinct sub-segment based on the Peano curve.

Quasi-simultaneous exposure of varying geometries is based on the repetitive, consecutive exposure of distinct fractal patterns. Each sub-segment constitutes a closed loop, allowing for an uninterrupted quasi-simultaneous exposure, schematically displayed in Figure 4.

**Figure 5:**
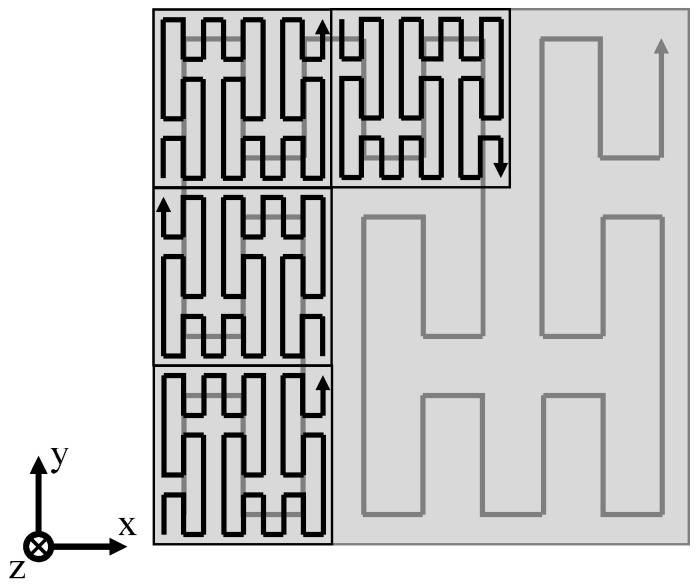
Schematic illustration of the fractal exposure sequence of distinct segments.

Quasi-simultaneous exposure is correlated with a significantly increased layer time due to the repetitive exposure of distinct segments, leading to an increase in the layer time equivalent to the additional number of exposure steps compared to single exposure. Distinct, repetitively exposed sub-segments are sequenced by applying a fractal exposure sequence to reduce geometry-induced influences and interferences on resulting temperature fields. Therefore, fractal patterns are applied on sub-segment level and for determining the sequence of consecutive segments. With regard to the scale-invariance of fractal space-filling curves, the sequence of consecutively scanned segments is determined by the structure of the Peano curve, schematically displayed in Figure 5.

Exposure patterns of complex cross-sections are generated applying the algorithm proposed by Yang et al. [26], thus implicitly generating a closed, space-filling curve corresponding to the specific part contour.

## 4. Materials and Methods

### 4.1. Experimental Set-Up

All experimental work is conducted using a freely configurable SLS research system, thus allowing for the integration of complex space-filling scan paths, prepared by means of iterative algorithms. The used SLS machine resembles commercially available machinery, equipped with a high-speed galvanometer scanner, SCANLAB GmbH, Puchheim, Germany, while allowing for the direct control of the exposure sequence based on the programming of exposure paths. Constant optical parameters include a laser wavelength of *λ* = 10.6 μm and a laser focus diameter of *d* = 0.5 mm. A layer height of 0.1 mm is kept constant for all experimental investigations. Commercially available Polyamide 12 powder, PA 2200, EOS GmbH, Krailling, Germany, is used as the underlying material. A mixture of equal fractions of pre-used powder and virgin powder is applied. The pre-used powder was extracted from overflow bins and was exposed to a single recoating step, corresponding to merely insignificant thermal aging. Resulting material properties exhibit a bulk density of 0.44 g cm^−3^ and a viscosity number, determined according to ISO 307, of *VN* = 64 mL g^−1^. The applied material exhibits a melting peak temperature of 179 °C and a crystallization peak temperature of 152 °C. To allow for thermographic investigations of exposure-induced temperature fields, a thermographic camera, VELOX 1310k SM, IRCAM GmbH, Erlangen, Germany, was used. Thermographic imaging was conducted using a frame rate of 355 Hz and a spatial resolution of 140 μm, covering a rectangular powder bed cross section of 40 × 40 mm^2^. An emission coefficient of *ε* = 0.9 was specified for determining resulting temperature fields.

### 4.2. Design of Experiments

The applied methodology is based on complementary thermographic and morphological investigations of components manufactured at non-isothermal processing conditions. Thermographic investigations are applied for characterizing thermal process properties of both conventional and quasi-simultaneous exposure strategies. For investigating processing properties of linear and fractal exposure strategies, single layers of square cross-sections exhibiting an edge length of 16.2 mm are fabricated. For enabling the comparison of linear and fractal exposure strategies at non-isothermal processing conditions, linear meander scanning and fractal scanning, corresponding to the Peano curve, are applied using a constant scan speed of 500 mm s^−1^, a laser power of 16 W, a hatch distance of 0.2 mm, and a powder bed temperature of 100 °C. A single exposure step is applied to allow for the comparative assessment of processing properties of conventional exposure strategies under non-isothermal processing conditions. The edge length specified for the thermographic investigation of single-scan exposure strategies is chosen according to the underlying hatch distance of 0.2 mm, corresponding to the 4th iteration of the Peano curve.

In addition to the application of single-step exposure strategies, thermographic investigations are conducted for characterizing thermal process properties of quasi-simultaneous, fractal exposure by varying the number of consecutive scans for determining the influence of quasi-simultaneous processing conditions on resulting transient thermal fields. Manufactured specimens compromise cubic geometries with an edge length of 10 mm, allowing for the investigation of the thermal superposition of distinct segments by means of thermographic analysis. Specimens are manufactured using a hatch distance of 0.2 mm and a laser power of 2 W. For characterizing the influence of varying quasi-simultaneous conditions and varying powder bed temperatures on emerging temperature fields, a full-factorial parameter variation is conducted, as displayed in Table 1. Varied process parameters include the number of scans, corresponding to the number of consecutive exposure steps of a distinct segment.

Complementary morphological investigations of manufactured specimens are based on applying constant quasi-simultaneous parameters and varied thermal boundary conditions, corresponding to a powder bed temperature of 75 °C and 100 °C, respectively. Based on preceding thermographic investigations, a number of 25 consecutive exposure steps are applied to ensure a homogenous layer formation. To allow for the comparison of thermal and morphological material characteristics in dependence of the underlying process, geometrically identical specimens are manufactured by applying quasi-isothermal PBF using a powder bed temperature of 170 °C, a hatch distance of 0.2 mm, a laser power of 16 W, and an exposure speed of 2000 mm s^−1^. Meander scanning is employed as the underlying exposure strategy.

### 4.3. Fabrication of a Complex Geometry Demonstrator

To investigate the capability of the proposed process strategy for the manufacturing of complex parts, a compression spring, exhibiting an outer diameter of 10 mm, a free length of 15 mm, and a wire diameter of 1 mm, is manufactured applying the Peano-based, quasi-simultaneous exposure strategy. In order to improve the manufacturing of thin-walled components with an increased surface-volume ratio, a quasi-simultaneous contour scan is applied subsequent to the hatch exposure. Applied processing parameters are displayed in Table 2.

A contour offset of 0.2 mm is specified, corresponding to the applied hatch distance.

### 4.4. Thermal and Morphological Characterization

Thermal characterizations of manufactured specimens are conducted using differential scanning calorimetry (DSC), applying a heating rate of 10 K min^−1^. Sample preparation for DSC measurements includes the removal of the edge region in order to allow for the comparability of varying processing conditions. Morphological characteristics are determined by applying both polarization microscopy and computed tomography. Polarized micrographs are prepared using thin sections of 10 μm thickness. Corresponding microscopic investigations are conducted using a Zeiss Axio Imager 2, Carl Zeiss Microscopy Deutschland GmbH, Oberkochen, Germany. Computed tomography of manufactured specimens is conducted using a sub-μ-CT, Fraunhofer Institute for Integrated Circuits (IIS) e.V., applying an isotropic spatial resolution of 4.5 μm. Subsequent analytical investigations of the spatial distribution of pores rely on script-based analysis of three-dimensional, binarized density distributions.

## 5. Results and Discussion

### 5.1. Limitations of Single-Scan Exposure Strategies

**Figure 6:**
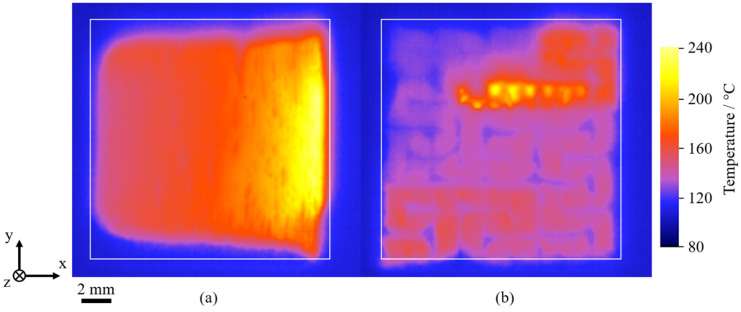
Thermographic imaging of the spatial distortion of the single-scan meander strategy (**a**) and the fractal Peano strategy (**b**) at a powder bed temperature of 100 °C, specified geometry indicated by a white square.

Considering processing properties of linear, conventional meander exposure at non-isothermal processing conditions, thermographic investigations of manufactured single layers exhibit considerable distortion following the exposure step. Displayed in Figure 6a, single layers manufactured using linear exposure paths depict a contraction perpendicular to the formed thermal gradient. Concerning the emergence of warping and curling of manufactured single layers, the manufacturing of parts without the application of support structures is inherently restricted due to build process interruptions. In contrast, the application of the fractal Peano strategy displays a considerably reduced extent of distortion, indicating an improved applicability of fractal exposure strategies for support-free non-isothermal processing.

Considering reduced peak temperatures observed for the application of fractal exposure strategies [29], a reduced thermal superposition of consecutively exposed scan paths is correlated with the observed, reduced distortion of manufactured layers. A reduced resulting distortion is in accordance with findings described by Ma et al. [27] and Catchpole-Smith et al. [28] for PBF-LB/M, indicating a significant reduction of residual stresses [27,28] and the occurrence of stress cracking [28] of nickel-base superalloys. Consequently, fractal exposure of distinct segments allows for the considerable reduction of stress-induced distortion, hence representing a foundation for the proposed approach of combined fractal, quasi-simultaneous exposure.

### 5.2. Thermographic Analysis of Fractal, Quasi-Simultaneous Processing

**Figure 7:**
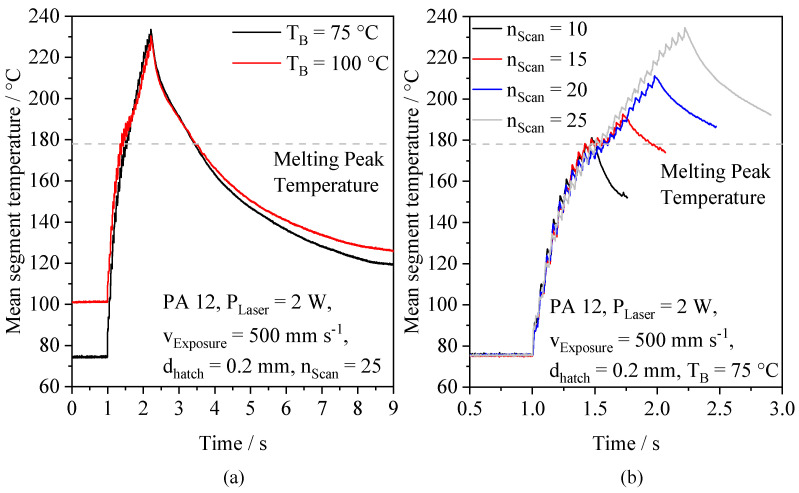
Influence of varying powder bed temperatures (**a**) and varying scan numbers (**b**) on resulting mean temperatures, occurring for the exposure of single, consecutively exposed segments.

The thermographic investigation of quasi-simultaneous exposure at non-isothermal conditions displays a characteristic gradual increase of observed mean segment temperatures. An increasing number of consecutive scans is correlated with a degressive increase, displayed in Figure 7a. Considering emergent peak temperatures, the excess of superficial melting of the powder bed significantly depends on the number of consecutive scans, being correlated with the applied energy density. Considering a degressive thermal increase, a similar thermal evolution can be observed for isothermal exposure using the meander exposure strategy due to a thermal superposition of consecutively exposed scan paths [19,20]. The influence of the applied powder bed temperature is clearly displayed with regard to the cooling rate, indicating a negative correlation of the applied temperature and the cooling rate. However, no significant influence of the powder bed temperature on resulting peak temperatures is observed, indicating a superposing influence of the local powder bed morphology on measured peak temperatures. Varying numbers of applied consecutive scans of distinct segments exhibit a quasi-linear relation of the number of applied scans and resulting peak temperatures, shown in Figure 7b, thus enabling the adjustment of resulting peak temperatures.

**Figure 8:**
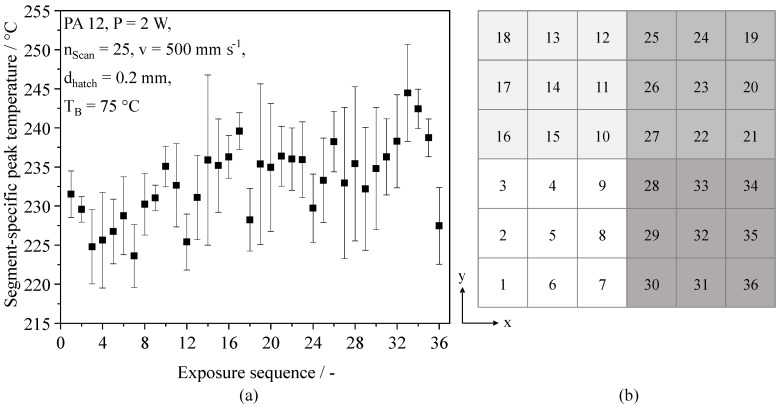
(**a**) Influence of the exposure sequence of a 10 × 10 mm^2^ cross-section on resulting peak temperature values; (**b**) Spatial representation of the fractal exposure sequence of distinct segments.

Considering the influence of thermal interdependencies of consecutively exposed segments on resulting residual stress in PBF-LB/M [22] and the occurrence of thermal interaction of subsequently exposed scan vectors, observed in PBF-LB/P [20], correlations of fractal sequencing on resulting peak temperatures are investigated. Measured segment-specific peak temperatures of square 10 × 10 mm^2^ cross-sections exhibit a merely insignificant influence of the exposure sequence, displayed in Figure 8.

Given the applied process parameters, fractal sequencing allows for the reduction of thermal superposition with regard to observed peak temperatures. Considering the fractal structure of the applied exposure sequence, a local, significant increase of peak temperatures can be observed, being correlated with the local meander-shaped sequence of the Peano curve. However, thermal superposition is predominantly limited to the local thermal interference of consecutively exposed segments, restricting the cross-component thermal superposition of exposed segments. Given the considerable reduction of part warping, a reduced thermal interdependence of distinct segments can be correlated with reduced stress-induced distortion, thus representing the foundation for the support-free, non-isothermal manufacturing of solid parts. Furthermore, with regard to the applied fractal sequencing of distinct segments, the observed merely local thermal superposition implies the possibility of limiting geometry-dependent influences on manufactured parts, hence allowing for the fabrication of parts with negligible variations of the local thermal history, regardless of underlying part geometries.

### 5.3. Part Morphology

**Figure 9:**
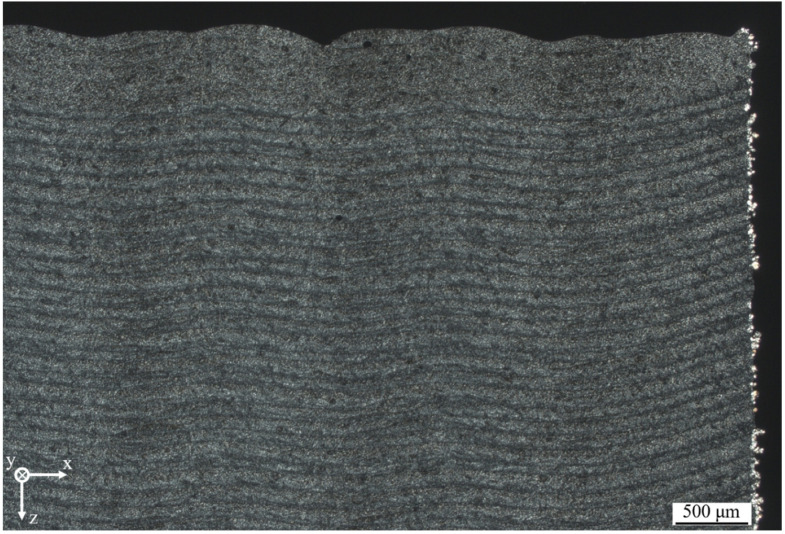
Polarization micrograph of a cubic specimen manufactured at non-isothermal conditions, d_thin-section_ = 10 μm.

Non-isothermal, quasi-simultaneous PBF of Polyamide 12 exhibits considerable morphological differences compared to components processed by means of isothermal PBF. Thin sections of manufactured cubic samples display a layer-wise crystallization of distinct layers, exhibiting a parabolic melt pool geometry of exposed segments, displayed in Figure 9.

Based on the displayed layer-wise structure, a dependence of the thermal penetration depth on the thermal superposition of distinct segments can be observed, leading to a reduced thermal penetration depth in the edge area. With regard to the observed, locally varying penetration depth, the previously observed relation of the number of consecutive exposure steps and the resulting peak temperature indicates a non-linear influence of the number of applied consecutive exposure steps. Considering the relation of the thermal diffusion length *μ*, the thermal impact time *t*, and the thermal diffusivity *α* [32], valid for the assumption of pulsed heating, a non-linear influence of a varying, exposure-dependent thermal impact time on the resulting thermal penetration depth is evident.

(4) $$\mu =2\sqrt{\alpha t}$$

In addition, the observed degressive increase of the segment-specific temperature indicates a superposing influence of the previously described relation of the number of scan paths and the resulting peak temperature on the emerging melt pool geometry. Consequently, the observed thickness of the top layer can be correlated with the applied quasi-simultaneous exposure, being induced by a considerable increased thermal impact time of the laser exposure compared to conventional exposure. 

**Figure 10:**
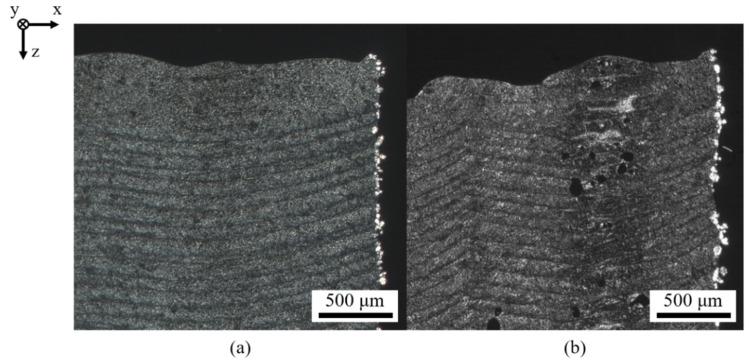
Polarization micrographs of cubic samples manufactured applying a powder bed temperature of 75 °C (**a**) and 100 °C (**b**).

With regard to variations of the applied powder bed temperature implicitly influencing transient temperature fields of distinct segments, an influence on the extent of thermal degradation can be observed, displayed in Figure 10.

Resulting morphologies depict a considerable influence of the applied powder bed temperature, affecting both the homogeneity of formed layers and the occurrence of thermal degradation in the center of distinct exposed segments. The extent of the occurrence of defects can be correlated with thermographic findings, showing a negative correlation of the applied powder bed temperature and the corresponding cooling rate, hence limiting thermal degradation. Furthermore, an increased powder bed temperature is correlated with the emergence of warping of manufactured parts, indicating the requirement of accelerated cooling of distinct exposed segments for increasing the geometric part accuracy.

**Figure 11:**
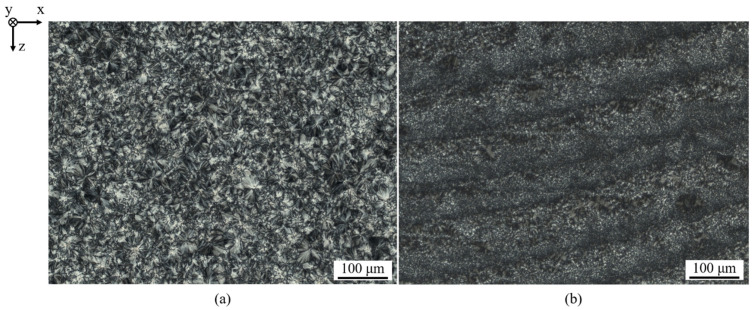
Polarization micrographs of Polyamide 12 specimens manufactured applying isothermal (T_B_ = 170 °C, v = 2000 mm s^−1^, P = 16 W, d_hatch_ = 0.2 mm) (**a**) and non-isothermal (T_B_ = 75 °C, v = 500 mm s^−1^, P = 2 W, d_hatch_ = 0.2 mm) (**b**) processing.

Varying spherulite sizes, formed within distinct layers, imply the occurrence of locally varying thermal conditions within the melt pool, leading to varying crystallization kinetics and resulting spherulite sizes [33]. Considering crystalline structures formed in isothermal PBF-LB/P, non-isothermal PBF-LB/P promotes the formation of significantly reduced spherulite diameters regardless of the position within a particular layer. Therefore, specimens manufactured by means of non-isothermal PBF exhibit a reduced morphological homogeneity resulting from locally varying cooling rates, displayed in Figure 11, which is considered inherently related to non-isothermal processing conditions [34]. In contrast to isothermal processing, no structural morphologic differences of edge regions are formed in parts produced by means of non-isothermal processing, indicating a potential improvement of superficial mechanical and tribological properties of manufactured components.

Considering the influence of locally varying cooling rates on resulting spherulite diameters [33], similar spherulite dimensions observed within the build plane indicate a predominant thermal homogeneity during the exposure of distinct segments.

**Figure 12:**
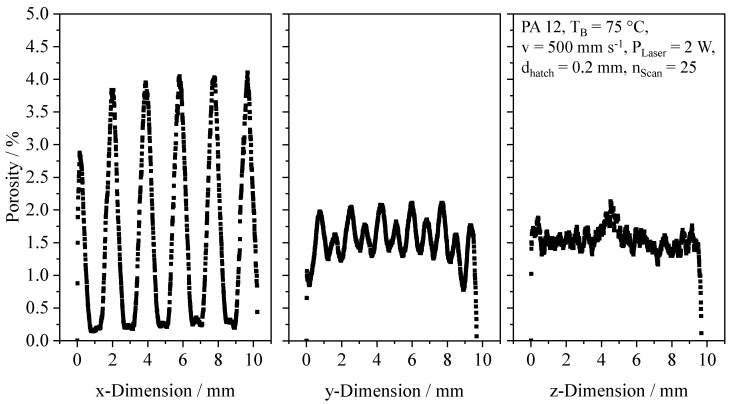
Spatial porosity distribution of a cubic sample, applying fractal sequencing of sub-segments, d_Smoothing_ = 0.2 mm.

Using computed tomography, the occurrence of local thermal degradation is predominantly limited to the center of distinct segments. The formation of spherical pores can be correlated with excessive energy input into the melt pool, indicating a reduced level of optimum energy density. However, considering an overall density of 98.51% ±0.31, the satisfactory manufacturing of dense components can be demonstrated. Resulting spatial porosity distributions exhibit a periodic occurrence of thermal degradation within the build plane. In contrast, the average porosity level exhibits a predominant invariance towards the build height, displayed in Figure 12.

The applied exposure sequence of distinct segments is implicitly represented in resulting porosity distributions, displaying a considerable variance of porosity observed in the YZ-plane. Observed anisotropic influences are correlated with the exposure sequence of consecutive segments. With regard to the applied exposure sequence locally resembling the meander strategy, a thermal superposition of consecutively exposed segments can be correlated with evenly distributed porosity, induced by thermal decomposition. In contrast, an increased time span between the exposure of segments located next to each other limits thermal decomposition in the edge region of distinct segments, leading to the formation of dense part regions. These findings are in good accordance with thermographic results, indicating a local thermal superposition, thus influencing the local emergence of thermal decomposition. Resulting from the segmented structure of the exposure strategy, observed morphological properties indicate an implicit compensation of the shrinkage of distinct segments due to the segmented build process. Corresponding implications on a potentially improved dimensional accuracy of manufactured parts will be addressed in future research.

Therefore, in contrast to quasi-isothermal PBF, non-isothermal PBF allows for manufacturing quasi-dense sections, exhibiting an anisotropic, lamellar morphology. However, the locally restricted formation of porosity implies the requirement for parameter optimizations to avoid thermal degradation.

### 5.4. Thermal Material Properties

**Figure 13:**
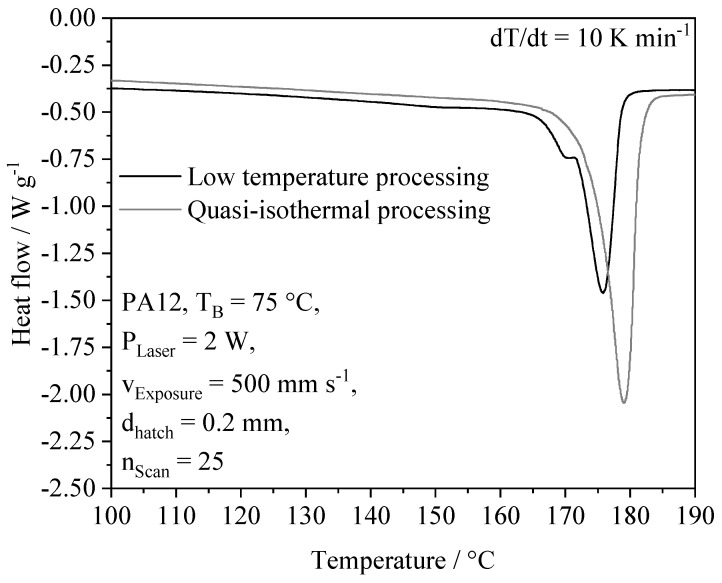
Differential scanning calorimetry of specimens manufactured using non-isothermal PBF and isothermal PBF.

Using differential scanning calorimetry, similar properties of components manufactured using isothermal and non-isothermal PBF-LB/P can be observed. In contrast to the isothermal crystallization of Polyamide 12 at reduced ambient temperatures, described by Paolucci et al. [14], no exothermal peak, correlated with cold crystallization, can be observed for the application of a powder bed temperature of 75 °C, displayed in Figure 13. Displayed results are in good accordance with findings described by Zhao et al. [13], exhibiting a negative correlation of the applied cooling rate and the melting peak temperature of fabricated samples, determined by means of DSC.

The absence of cold crystallization indicates the predominant crystallization above the applied powder bed temperature, which is in accordance with thermographic investigations, showing a slow cooling process compared to thermal quenching, applied by Paolucci et al. [14]. Considering a melting enthalpy of −53.41 J/g ± 1.78 and −61.95 J/g ± 1.35 for Polyamide 12 processed at non-isothermal conditions and by means of quasi-isothermal processing, respectively, structural differences can be determined, indicating a slightly reduced crystallinity. Furthermore, a reduced melting point of specimens prepared at non-isothermal conditions is evident. These observations are in accordance with findings described by Kigure et al. [34] for the non-isothermal processing of Polyamide 12 using support structures, showing a comparable reduction in the degree of crystallinity and the melting peak temperature. Considering the applied powder bed temperature, no significant influence of an elevated powder bed temperature of 130 °C, described by Kigure et al. [34], can be observed. Therefore, the formation of similar thermal properties indicates a subordinate influence of the powder bed temperature on formed crystal modifications and the degree of crystallinity.

However, despite observed marginal variations in thermal material properties, non-isothermal processing promotes the formation of similar thermo-structural properties, correlated with similar crystalline structures, compared to the application of quasi-isothermal PBF-LB/P.

### 5.5. Manufacturing of Complex Geometries

**Figure 14:**
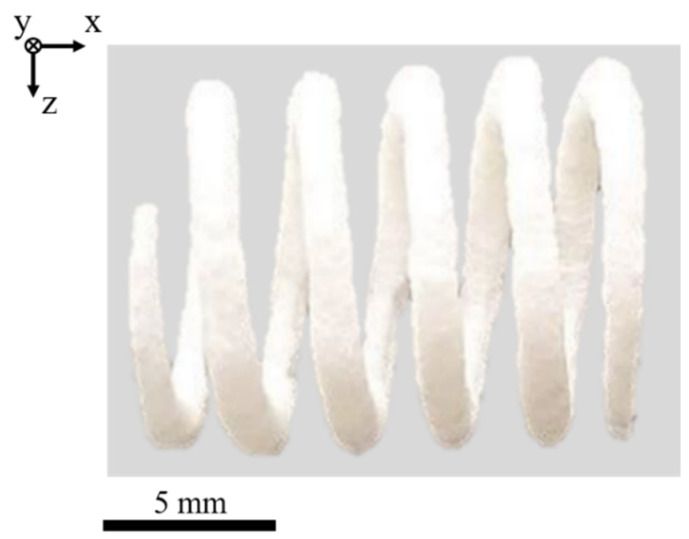
Depiction of a compression spring, manufactured by means of support-free low temperature PBF, T_B_ = 75 °C.

The applicability of low temperature PBF of complex geometries is correlated with varying layer times [3], implying varying cooling times. Manufactured compression springs, displayed in Figure 14, demonstrate the suitability of the proposed fractal, non-isothermal processing strategy for successfully manufacturing geometries with varying cross-sections at non-isothermal processing conditions. Consequently, the proposed approach allows for the manufacturing of thick-walled and thin-walled components based on the combination of locally quasi-simultaneous exposure and fractal scan path generation. With regard to the geometric accuracy, structural restrictions can be observed.

Resulting geometric deviations are correlated with the previously discussed elevated layer thickness, induced by the application of quasi-simultaneous exposure and an implicitly elevated thermal impact time. Consequently, optimizations of applied processing parameters and underlying thermal boundary conditions embed the potential for considerably increasing the geometric accuracy in future research.

## 6. Conclusions and Outlook

Within the present paper, a novel approach for the laser-based powder bed fusion of polymers was proposed. Based on the combination of fractal exposure strategies and quasi-simultaneous exposure of distinct segments, the non-isothermal, support-free manufacturing of varying part geometries could be demonstrated. Based on thermographic imaging, a gradual temperature increase of discrete segments of the exposed cross-section was identified. In combination with a subsequent immediate crystallization of distinct segments, the spatially and temporally discrete exposure constitutes the methodological foundation of non-isothermal processing. By applying considerably reduced powder bed temperatures, a lamellar morphology was obtained, exhibiting significantly reduced, locally varying spherulite sizes. Based on the segmented exposure strategy, shrinkage of discrete segments is limited to the mesoscopic level, allowing for the manufacturing of dense components.

Future research will focus on investigating mechanical properties of parts manufactured using low-temperature powder bed fusion, the optimization of process parameters in combination with the targeted local modification of the part morphology, as well as the integration of thermally sensitive additives, such as medically active substances, into manufactured parts. Furthermore, reducing the necessary number of consecutive exposure steps is essential for decreasing the layer time for enhancing the economic viability of low temperature PBF-LB/P. Therefore, potentials of low-temperature PBF-LB/P include the application of polymer blends with divergent thermal properties, considerably reduced thermal aging of polymers, and the manufacturing of thermally sensitive multi-material components.

## Figures and Tables

**Table 1:** Variation of processing parameters applied for thermographic investigations of non-isothermal PBF.

Number of Scans	Powder Bed Temperature
10, 15, 20, 25	75 °C, 100 °C

**Table 2:** Exposure parameters of the fractal, quasi-simultaneous exposure of thin-walled components.

Number of Consecutive Contour Scans	Number of Consecutive Hatch Scans	Powder Bed Temperature	Laser Power	Exposure Speed
50	25	75 °C	2 W	500 mm s^−1^

## Data Availability

The data presented in this study are available on request from the corresponding author. The data are not publicly available due to ongoing research in this field.
